# Combining Exosomes Derived from Immature DCs with Donor Antigen-Specific Treg Cells Induces Tolerance in a Rat Liver Allograft Model

**DOI:** 10.1038/srep32971

**Published:** 2016-09-19

**Authors:** Ben Ma, Jing-Yue Yang, Wen-jie Song, Rui Ding, Zhuo-chao Zhang, Hong-chen Ji, Xuan Zhang, Jian-lin Wang, Xi-sheng Yang, Kai-shan Tao, Ke-feng Dou, Xiao Li

**Affiliations:** 1 Department of Hepatobiliary Surgery, Xijing Hospital, Fourth Military Medical University, Xi’an, Shaanxi Province, China; 2 Department of Clinical Oncology, Xijing Hospital, Fourth Military Medical University, Xi’an, Shaanxi Province, China

## Abstract

Allograft tolerance is the ultimate goal in the field of transplantation immunology. Immature dendritic cells (imDCs) play an important role in establishing tolerance but have limitations, including potential for maturation, short lifespan *in vivo* and short storage times *in vitro*. However, exosomes (generally 30–100 nm) from imDCs (imDex) retain many source cell properties and may overcome these limitations. In previous reports, imDex prolonged the survival time of heart or intestine allografts. However, tolerance or long-term survival was not achieved unless immune suppressants were used. Regulatory T cells (Tregs) can protect allografts from immune rejection, and our previous study showed that the effects of imDex were significantly associated with Tregs. Therefore, we incorporated Tregs into the treatment protocol to further reduce or avoid suppressant use. We defined the optimal exosome dose as approximately 20 μg (per treatment before, during and after transplantation) in rat liver transplantation and the antigen-specific role of Tregs in protecting liver allografts. In the co-treatment group, recipients achieved long-term survival, and tolerance was induced. Moreover, imDex amplified Tregs, which required recipient DCs and were enhanced by IL-2. Fortunately, the expanded Tregs retained their regulatory ability and donor-specificity. Thus, imDex and donor-specific Tregs can collaboratively induce graft tolerance.

Life-long use of immune suppressants is necessary in organ transplantation to suppress immune rejection and prolong allograft survival. However, non-specific effects of these suppressants lead to an increased occurrence of opportunistic infection and cancer recurrence. Donor-specific tolerance, if established effectively, could avoid the use of these suppressants and eliminate these side effects.

Immature dendritic cells (imDCs) serve as immune system sentinels by sensing environmental and inflammatory signals, and they play an essential role in the maintenance of immune tolerance. In particular, imDCs play a key role in dictating the outcome of immune responses by influencing the balance between the inflammatory response and the regulatory T cell (Treg) response. At the centre of this immunological balance is finely regulated crosstalk between DCs and Tregs, whereby Tregs modulate DC phenotypes and functions and DCs drive Tregs differentiation. ImDCs and Tregs alone or in combination can effectively prolong allograft survival time^1^,^2^,^3^. However, DC-based technology has deficiencies that limit its application in the clinic, including potential maturation, short-term lifespan *in vivo*, a seven-day production requirement, and the inability to be preserved long-term *in vitro*^4^,^5^. DCs can release small (approximately 30–100 nm in diameter) membrane-enclosed vesicles (exosomes) derived from the internal vesicles of multivesicular bodies (MVBs) into the extracellular milieu, which have been proposed to play an efficient role in antigen presentation, immune regulation and signal transduction.

Some reports, including our previous study, revealed that the administration of donor exosomes from imDCs (imDex) before transplantation prolonged the survival time of heart or intestine allografts^6^,^7^,^8^. However, it is not clear whether imDex could work in liver transplantation. Moreover, immune tolerance or long-term graft survival was not achieved in past reported treatment protocols with imDex unless immune suppressants were used at the same time. Tregs are known to protect skin, cardiac and renal allografts from immune rejection^9^,^10^ and the effects of imDex *in vivo* demonstrated a close relationship with Tregs in our previous study as well as others’^4^,^8^,^11^. Therefore, we incorporated Tregs into the combined treatment protocol to further reduce or avoid the use of suppressants. There are on-going controversies as to whether donor antigen specificity is needed for Tregs in transplantation immune regulation. Thus, we treated recipients with different dosages of donor-derived imDex and/or different donor-specific Tregs and observed the immune regulatory effects *in vitro* and *in vivo*.

## Results

### DEX isolation and identification

**Figure 1:**
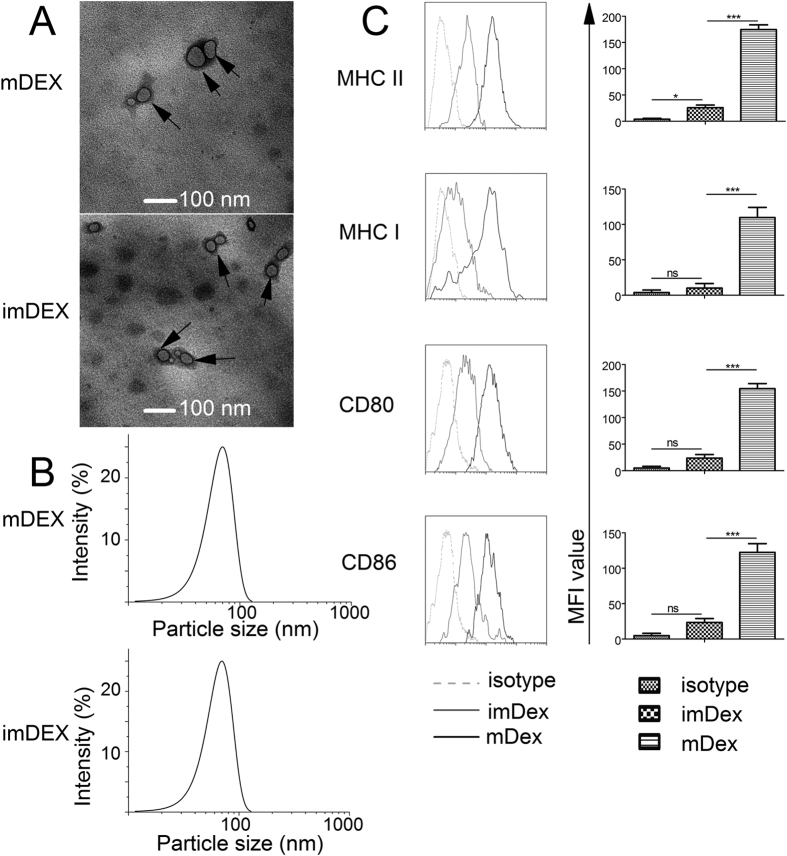
Characterization of exosomes derived from DCs. (**A**) Two types of exosomes were observed by electron microscopy. Bar represents 100 nm. Arrows indicate exosomes. Representative fields are shown. (**B**) Particle size of exosomes (mDex is 73.8 ± 20.8 nm; imDex is 69.9 ± 18.0 nm). (**C**) The phenotypic profiles of imDex and mDex were analysed by cytofluorometry. The results shown are representative of three independent experiments. (**D**) Mean fluorescence intensity (MFI) was analysed. *Indicates *p* < 0.05; ***indicates *p *< 0.001; ns indicates no significant difference.

We obtained exosomes from imDCs (imDex) and from mDCs (mDex) as described in the Methods. Electron microscopy results of exosomes revealed typical image of heterogeneous vesicles (30–120 nm in diameter)^12^ (Fig. 1A,B). The exosomes were analysed by FCM (flow cytometry), and typical imDex and mDex phenotypic profiles are shown in Fig. 1C/D. Lower levels of CD80, CD86, MHC class I and MHC class II were expressed on imDex than on mDex (compared with mean fluorescence intensity, in Fig. 1D). These exosomes were in agreement with those described in previous reports^8^,^11^.

### Obtaining and identifying donor-specific Tregs

**Figure 2:**
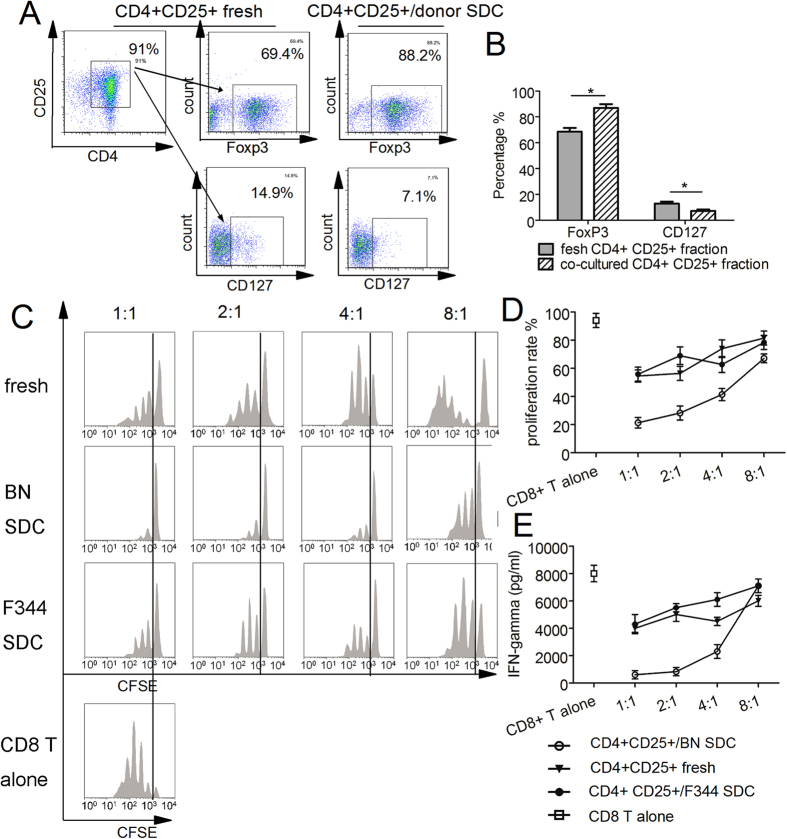
Obtaining and identifying donor antigen-specific Tregs. (**A**) Both fresh isolated CD4^+^ CD25^+^ T cells and donor SDC co-cultured CD4^+^ CD25^+^ T cells were analysed by FCM. (**B**) The FOXP3 and CD127 expression rates in the two fractions of CD4^+^ CD25^+^ T cells were analyzed. (**C,D**) Graded numbers of pre-activated CD4^+^ CD25^+^ cells or fresh CD4^+^ CD25^+^ T cells were added to CFSE-labelled CD8a+ T cells as described in the Methods (n = 3 at each ratio). (**C**) CFSE was assessed by FCM, and (**D**) the proliferation rate was analysed. (**E**) IFN-γ was measured from the culture supernatants of the mixed culture system. One representative of three independent experiments is shown in (**A**/**C**). *Indicates *p* < 0.05.

CD4^+^ CD25^+^ T cells were sorted by magnetic isolation from the spleens of Lewis rats. CD4^+^ CD25^+^ cells comprised 89.1 ± 2.9% of this fraction, as shown in Fig. 2A/B and Supplementary Table S1. When gated on CD4^+^ CD25^+^ cells (Fig. 2A), FOXP3^+^ cells were 68.5 ± 2.9%, and CD127^+^ cells were 12.9 ± 1.4% (Fig. 2A/B and Supplementary Table S1). The CD4^+^ CD25^+^ cells from Lewis rats were co-cultured with IL-2 and 1 × 10^6^ γ-irradiated (20 Gy, 2,000 rad) SDCs (dendritic cells isolated from spleen) from Brown Norway (BN) rats for 14 d. The co-cultured CD4^+^ CD25^+^ T cells, which often expanded 2- to 5-fold, were prepared for FCM analysis. Gated on CD4^+^ CD25^+^ cells, the FOXP3 expression rate was 86.9 ± 3.0% (Supplementary Table S1) (increased from the previous CD4^+^ CD25^+^ fraction, *p* = 0.0114, n = 3, Fig. 2A/B), and CD127 expression was 7.2 ± 1.0% (Supplementary Table S1) (decreased from the fresh isolated CD4^+^ CD25^+^ fraction, *p* = 0.0334, n = 3, Fig. 2A/B). The co-cultured CD4^+^ CD25^+^ T cells were similar to those described in previous reports in mice and humans^13^,^14^. To further clarify the status of these two fraction cells, we also detected CTLA-4, Ki67 and ICOS (Supplementary Fig. S1) and found co-cultured CD4+ CD25+ T cells expresses those molecules (expressed CTLA-4, Ki67 and ICOS, meanwhile with higher expression of Ki67 compared with fresh isolated CD4+ CD25+ cells, *p* < 0.05, n = 3), which suggested that this co-cultured CD4^+^ CD25^+^ cells might be active and had the potential for immune regulation. They were then ready for therapeutic administration.

Functional assays were performed with pre-activated CD4^+^ CD25^+^ cells as suppressors, CFSE-labelled Lewis CD8a^+^ T cells as responders, and BN SDCs as stimulators. As the amount of CD4^+^ CD25^+^ T cells decreased, the proliferation of CD8a^+^ T cells increased, which suggested a dose-dependent suppression of CD8a^+^ T cell proliferation (Fig. 2C/D). This suppression was donor-specific, as dose-dependent suppression was not seen when the suppressors were F344 SDC pre-activated CD4^+^ CD25^+^ T cells or fresh isolated CD4^+^ CD25^+^ T cells.

To further clarify this finding, we measured the production of IFN-γ and found that IFN-γ was similarly inhibited by the pre-activated CD4^+^ CD25^+^ T cells in a dose-dependent and donor-specific manner (Fig. 2E). Thus, we had obtained Tregs with donor antigen-specific suppression activity (donor-specific Tregs)^15^,^16^.

### Administration of donor imDex in combination with donor-specific Tregs prolonged liver allograft survival

**Figure 3:**
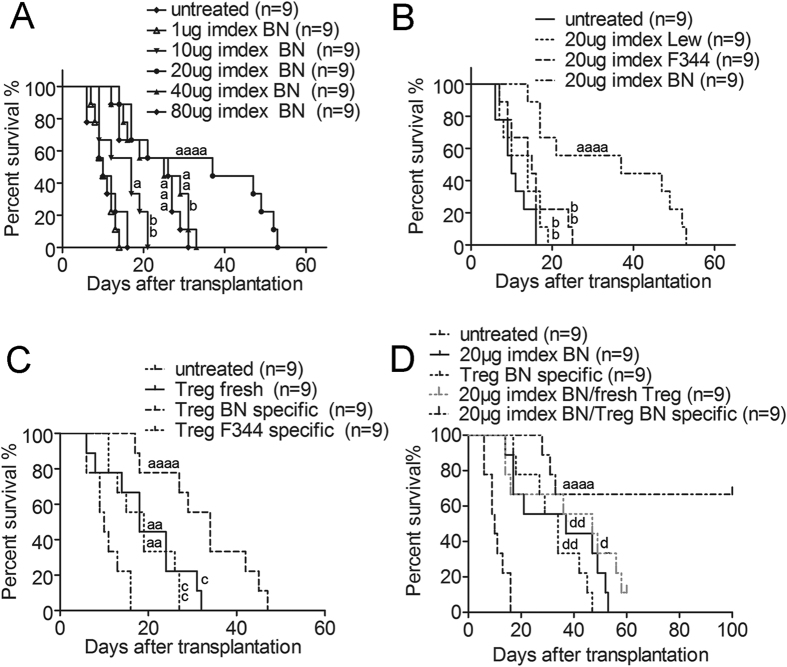
Administration of donor imDex, different Tregs or both combined promotes liver allograft survival. (**A**) The percentage of surviving liver allografts with different dosages of donor imDex. (**B**) ImDex derived from donors (BN), recipients (Lewis), or other allogeneic donors (F344) were injected at 20 μg into recipient rats. (**C**) Recipients were treated with different Tregs. (**D**) The liver allograft survival with the administration of 20 μg imDex and/or BN-specific/fresh Tregs. ^a^Compared with untreated group; ^b^compared with 20 μg BN derived imDex treatment group; ^c^compared with BN-specific Tregs treatment group; ^d^compared with 20 μg imDex plus BN specific Tregs treatment group. One a/b/c/d, *p* < 0.05; two a/b/c/d, *p* < 0.01; three a/b/c/d, *p* < 0.001; four a/b/c/d, *p* < 0.0001.

We first analysed the effects of different doses (1, 10, 20, 40 or 80 μg) of imDex injected via the caudal vein 7 d before, the day of, and 7 d after transplantation (Fig. 3A). The 10, 40 and 80 μg group survival times were significantly longer than the untreated group (MST: 17 d for 10 μg, *p* = 0.0241, n = 9; 25 d for 40 μg, *p* = 0.0008, n = 9; 26 d for 80 μg, *p* = 0.0011, n = 9). However, the longest allograft survival time was obtained in the 20 μg group (MST: 37 d, n = 9), and it was significantly increased compared with the 10 μg group (*p* = 0.0087, n = 9), 40 μg group (*p* = 0.0498, n = 9) and 80 μg group (*p* = 0.0441, n = 9) (Fig. 3A/Table 1), indicating that 20 μg (per treatment of three) was the optimal dosage.

We tested the effects of 20 μg of imDex derived from BN, F344 or Lewis rats. Only imDex derived from donor rats significantly prolonged the recipient survival times compared with the untreated group (MST: 14 d for Lewis, *p* = 0.1754 vs. untreated group, n = 9; 15 d for F344, *p* = 0.1159 vs. untreated group, n = 9; Fig. 3B/Table 1), indicating that the effect was donor specific.

Three types of Tregs (Lewis-derived fresh Tregs, Lewis-derived BN-specific Tregs, and Lewis-derived F344-specific Tregs) were injected into recipients at a dose of 2 × 10^6^ via the caudal vein 7 d before, the day of, and 7 d after transplantation (Fig. 3C/Table 1). The recipient survival times in the BN antigen-specific Tregs-treated group were longer than in the untreated groups (MST: 34 d vs. 10 d, *p* < 0.0001, n = 9). The F344 antigen-specific Tregs and fresh Tregs also prolonged graft survival times compared with the untreated group (MST: fresh Tregs group vs. untreated group = 18 d vs. 10 d, *p* = 0.0081, n = 9; MST: F344-specific group vs. untreated group = 19 d vs. 10 d, *p* = 0.006, n = 9), but not to the same degree (fresh Tregs group vs. BN-specific group, *p* = 0.01, n = 9; F344-specific group vs. BN-specific group, *p* = 0.0032, n = 9), suggesting that Tregs prolong graft survival time with donor specificity.

To further improve allograft survival time, we co-injected 20 μg of donor imDex with donor-specific Tregs, as imDCs have been reported to work with Tregs *in vivo* and *in vitro*^8^,^17^,^18^,^19^. While the MSTs of recipients from the imDex and Tregs-alone treatment groups reached 37 d and 34 d, respectively, the combination of donor imDex with donor-specific Tregs induced the long-term survival of liver allografts (6 recipients survived over 100 d, *p* = 0.007 compared with the 20 μg imDex group, n = 9; *p* = 0.0083 compared with the BN-specific Tregs group, n = 9) (Fig. 3D/Table 1). Furthermore, we also combined donor imDex with fresh isolated Tregs as a control group, and the MST of this group reached 47 d (*p* = 0.0386, n = 9, compared with the experimental group; *p *= 0.283, n = 9, compared with the 20 μg imDex group) (Fig. 3D/Table 1), which suggested that it matters that the combined Tregs were donor-specific, for the extended survival time in the experimental group compared with the imDex alone group.

### Infiltrating cells and rejection symptoms in allografts were reduced

**Figure 4:**
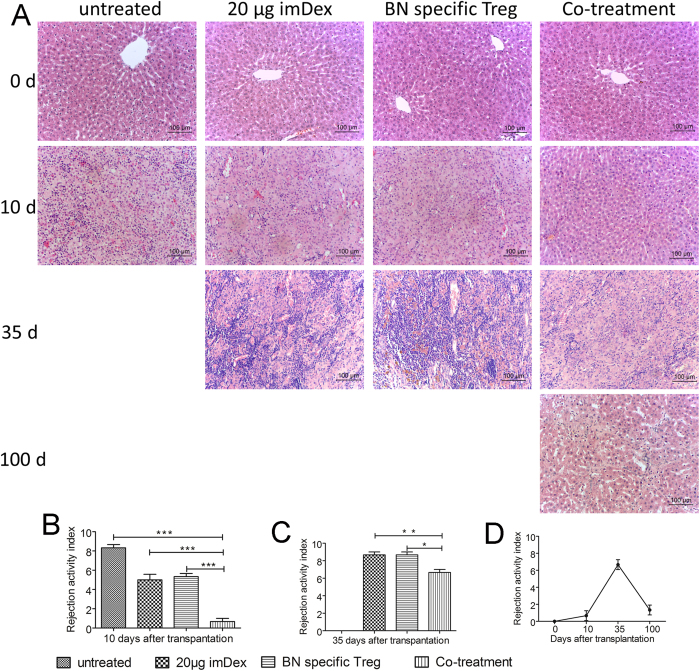
Pathology of rat liver grafts in each group (magnification: ×200). (**A**) On the 10^th^, 35^th^ and 100^th^ days after liver transplantation, three recipient grafts were harvested in each group and stained with HE for pathological analysis (8 × 200 fields per slide and 3 slides per animal. Representative fields are shown in A). Due to the relatively short survival times, grafts from some groups are not shown for the 35 and 100 day analyses. Bars represent 100 μm. (**B**) Analysis of RAI among the 10-day grafts. (**C**) Analysis of RAI among the 35-day grafts. (**D**) RAI analysis among the co-treatment group grafts on the 10^th^, 35^th^ and 100^th^ day after liver transplantation. *Indicates *p* < 0.05; **indicates *p* < 0.01; ***indicates *p* < 0.001.

To evaluate the degree of allograft rejection, we harvested grafts on days 0, 10, 35 and 100 after transplantation. The rejection activity index (RAI) according to the Banff schema was used as the standard to evaluate rejection severity in each group. Haematoxylin and eosin (HE) staining showed that the levels of infiltrating inflammatory cells varied in different groups and on different days (Fig. 4A). The 0-day grafts from each group showed normal results.

On the 10^th^ day, the graft pathology results showed different degrees of degeneration and necrosis in liver parenchyma cells in the four groups. HE staining of the untreated group revealed obvious inflammatory cell infiltration, hepatic lobule structural disorder and severe intravascular dermatitis (Fig. 4A). The RAI was 8.3 ± 0.3, and the rejection was Severe (Fig. 4B). Recipients in the 20 μg imDex treatment group and the donor-specific Tregs treatment group (Fig. 4A/B) showed similar lymphocytic infiltration in portal areas; the Banff classification was Moderate (RAI of the imDex group was 5.0 ± 0.6 and of the Tregs group was 5.3 ± 0.3). The 20 μg BN imDex/BN-specific Tregs co-treatment group (Fig. 4A/B) exhibited a very small amount of mononuclear cell infiltration and the Banff grade was Undefined. The RAI (0.7 ± 0.3) was significantly reduced (Fig. 4B) compared with the other three groups (untreated group: *p* < 0.001, n = 3; imDex treated group: *p* < 0.001, n = 3; donor-specific Tregs group: *p* < 0.001, n = 3).

On the 35^th^ day, a large number of infiltrating cells and chronic rejection symptoms such as biliary atresia and cholestasis appeared in the imDex and Tregs treatment groups. However, no chronic rejection symptoms appeared in the co-treatment group (Fig. 4A) on the 35^th^ day. In this group, inflammatory cell infiltration into the grafts was reduced compared with the imDex or Tregs treatment groups. The RAI was 6.7 ± 0.3 (co-treatment vs. imDex alone, *p *< 0.01, n = 3; co-treatment vs. Tregs alone, *p *< 0.05, n = 3) (Fig. 4C). These results showed that 20 μg of BN imDex combined with BN-specific Tregs could reduce immune rejection in liver allografts.

On the 100^th^ day, the co-treatment group grafts exhibited fibrous regeneration and hepatic lobule structural disorder, but mononuclear cell infiltration was reduced (Fig. 4A). The Banff grade was Undefined, and the RAI was 1.3 ± 0.3, which was significantly reduced (Fig. 4D) compared with the 35-day grafts (RAI = 6.7 ± 0.3, *p *< 0.001, n = 3). The 10-, 35- and 100-day pathology changes suggested that co-treatment reduced rejection and helped receipt livers regenerate after undergoing slight acute rejection.

### Recipient immune responses were suppressed in a donor-specific manner

To explore the immune statue in recipients, we assessed the anti-donor cellular response in recipients after transplantation. As described in the Methods, 5 × 10^4^ purified T cells (isolated from splenocyte harvested 10 d after transplantation from recipients) were incubated with 5 × 10^4^ irradiated SDC (stimulator cells) derived from either donors (BN) or other allogeneic donor (F344).

**Figure 5:**
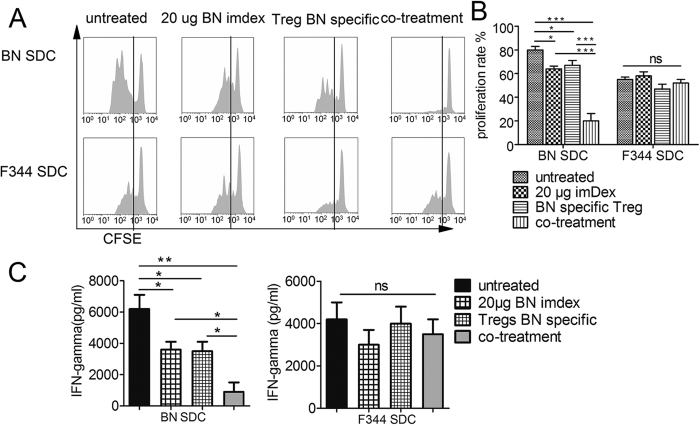
Analysis of the anti-donor cell response in recipients. As described in Methods, ten days after transplantation, 5 × 10^4 ^T cells purified from recipient splenocytes (from untreated recipients, 20 μg imDex treated recipients, BN-specific Tregs treated recipients or imDex/Tregs co-treated recipients; n = 3 in each group) were labelled with CFSE and incubated with equal numbers of irradiated SDCs (BN/F344 derived). (**A**) On day 5, CFSE intensity was measured and (**B**) the percentages of divided T cells were analysed. (**C**) IFN-γ was measured from culture supernatants on day 5, as described in the Methods. *Indicates *p* < 0.05; **indicates *p* < 0.01; ***indicates *p* < 0.001; ns indicates no significant difference.

As shown in Fig. 5A/B, total T cells from co-treated rats displayed a significant decrease in proliferation against BN SDCs compared with T cells from untreated recipients (*p* < 0.001, n = 3), 20 μg BN imDex-treated recipients (*p* < 0.001, n = 3) and BN-specific Tregs-treated recipients (*p* < 0.001, n = 3). However, total T cells from all four groups proliferated almost equally with the F344 SDCs used as stimulator cells (Fig. 5A/B). Furthermore, we observed that the IFN-γ levels in the T cell/donor SDC co-culture supernatant were similarly inhibited in a donor-specific manner (co-treatment group vs. untreated group: *p* < 0.01, n = 3, BN SDCs as stimulators, Fig. 5C; co-treatment group vs. untreated group: *p* > 0.05, n = 3, F344 SDCs as stimulators, Fig. 5C). These results demonstrated that the immune response of T cells in co-treated recipients was inhibited.

### Tregs distribution in the recipients

**Figure 6:**
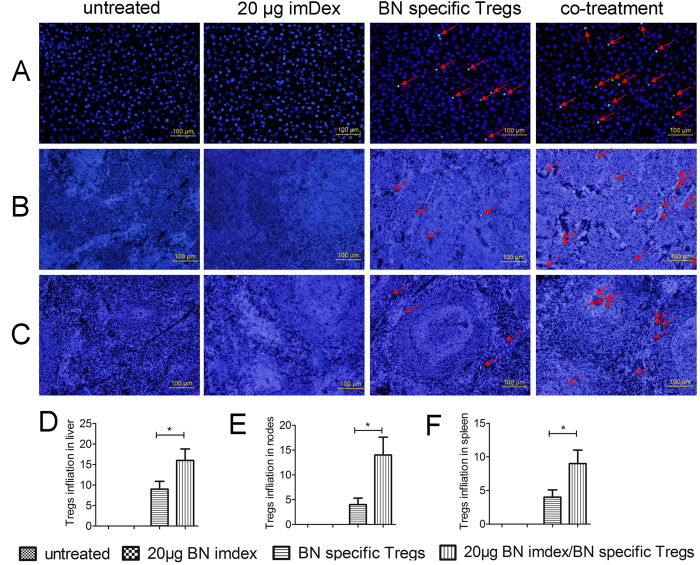
Immunofluorescence of liver grafts, mesenteric lymph nodes and spleens (magnification: ×200). Additional 12 recipients accepted no treatment, 20 μg imDex, BN-specific Tregs or imDex/Tregs co-treatment and each group contained 3 recipients. Samples were harvested 10 d after transplantation, for immunofluorescence. CFSE-labelled Tregs (green) were stained with DAPI (blue) and merged into cyan. Nuclei of parenchymal cells were stained blue with DAPI. The average number of positive cells was analysed in 8 random fields per slide (×200, 2 slides per animal, 3 animals per group, 10 days after transplantation). Bars represent 100 μm. (**A**) Liver grafts. (**B**) Mesenteric lymph nodes. (**C**) Spleen (one representative field is presented). (**D**–**F**) Analysis of the average number of CFSE-labelled Tregs in (**D**) liver grafts, (**E**) mesenteric lymph nodes and (**F**) spleens. One representative field of 48 random ×200 fields per group is shown. *Indicates *p* < 0.05.

To study the distribution of Tregs *in vivo*, we designed an independent test with CFSE-labelled Tregs. Twelve extra recipients accepted liver transplantation and four different treatments in the same dosage regimen described above, except that CFSE-labelled Tregs were used. On the 10^th^ day after transplantation, liver grafts, mesenteric lymph nodes and spleens were harvested for immunofluorescence. As shown in Fig. 6A–C, CFSE-labelled Tregs were distributed in these organs in the BN-specific Tregs treatment group and the co-treatment group. In liver grafts, we found that the number of CFSE^+^ cells in the co-treatment group was significantly higher than in the BN-specific Tregs treatment group (*p* = 0.0413, n = 48, Fig. 6D). In mesenteric-draining lymph nodes and spleens, similar results were observed (lymph nodes, *p* = 0.0105, n = 48; spleens, *p* = 0.0310, n = 48; Fig. 6E/F). Therefore, imDex might promote the proliferation of Tregs, which will enhance regulatory effects of Tregs *in vivo*, similar to imDCs^17^,^19^.

### Exploring the mechanism of the synergistic effects of donor imDex and donor-specific Tregs with *in vitro* and *in vivo* assays

We hypothesized that the infused exosomes were the reason why CFSE-labelled Tregs were increased in the co-treatment group. To verify this, we implemented *in vitro* Tregs proliferation assays. We used 2 × 10^4^ Lewis SDCs as “assistance cells” and 200 units/ml IL-2 as an “assistance cytokine”.

**Figure 7:**
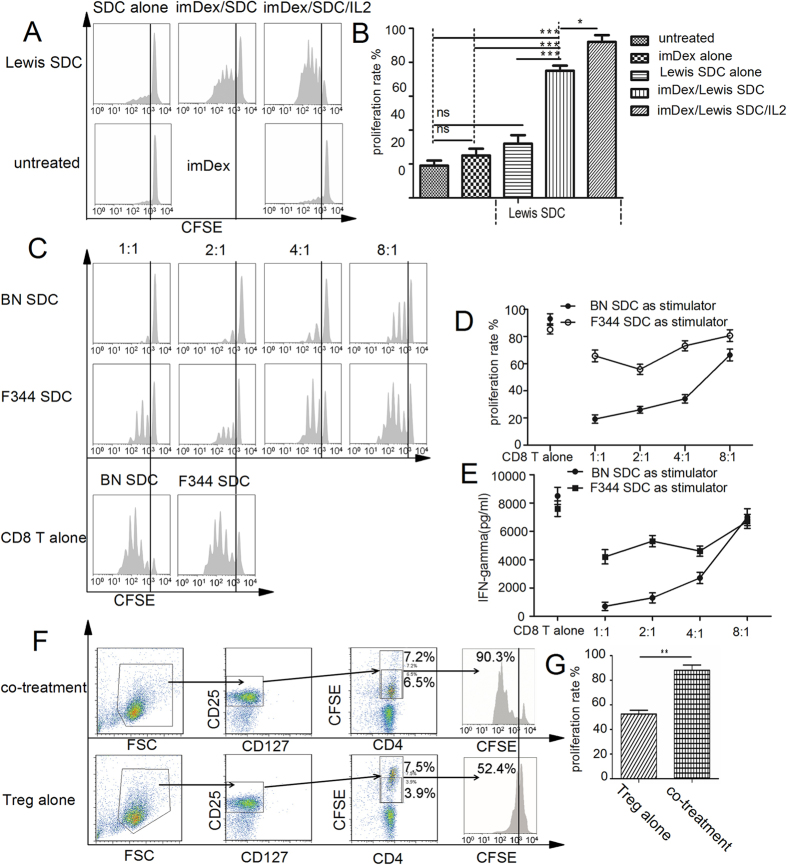
Donor-specific Tregs proliferation assays *in vitro* and *in vivo*. (**A**) In the Tregs expansion assay, as described in the Methods, 1 × 10^4^ Lewis-derived BN-specific Tregs (CFSE-labelled) were cultured with 0.5 μg BN imDex alone, 2 × 10^4^ Lewis SDCs, 0.5 μg BN imDex/Lewis SDCs or 0.5 μg BN imDex/Lewis SDCs/200 units/ml IL-2. Seven days later, CFSE intensity was measured by FCM, and representative results are shown. (**B**) The percentage of divided CFSE-labelled Tregs was analysed. (**C**) In the Tregs suppression assay, graded numbers of the imDex expanded Tregs were added to CFSE-labelled CD8a^+^ T cells as described in the Methods (n = 3 at each ratio). CFSE intensity was measured by FCM, and representative results are shown. (**D**) The percentage of divided CD8a^+^ T cells was assessed. (**E**) IFN-γ was measured and analysed in the above mixed-cell system. (**F**) In the *in vivo* assays, CD4^+^ CD25^+^T cells were isolated 10 d after transplantation from recipient splenocytes (BN-specific Tregs treatment or 20 μg donor imDex/BN-specific Tregs treatment group). Gated on CD4^+^ CD25^+^ CD127^−^ CFSE^+^ cell subsets (representative FACS gates and results are shown in F), (**G**) the percentage of divided CFSE-labelled Tregs was analysed. *Indicates *p* < 0.05; **indicates *p* < 0.01; ***indicates *p* < 0.001; ns indicates no significant difference.

As shown in Fig. 7A, no considerable proliferation was observed in the untreated group, imDex group or Lewis SDC group. However, Treg proliferation was increased in the imDex/Lewis SDC group compared to the imDex group (*p* < 0.001, n = 3) or the Lewis SDC group (*p* < 0.001, n = 3), indicating that imDex can amplify Tregs *in vitro* and that DCs are essential for this effect. When IL-2 was added to the administration protocol, Treg proliferation increased compared to the imDex/Lewis SDC group (*p < *0.05, n = 3), suggesting that IL-2 improved the ability of imDex to expand Tregs.

Next, we performed Treg suppression assays with the expanded Tregs. A dose-dependent suppression of CD8a^+^ T cell proliferation is shown in Fig. 7C/D. The expanded Tregs were still donor-specific, as dose-dependent suppression was not seen when F344 SDCs were used as stimulators (Fig. 7C/D). In the IFN-γ inhibition assays, we observed similar results (Fig. 7E). These results suggested that imDex-expanded Tregs maintain their regulatory ability and do not lose donor specificity.

ImDex was injected into Lewis rats via the caudal vein along with CFSE-labelled Tregs to detect proliferation of exogenous Tregs *in vivo*. On the 10^th^ day after transplantation, CD4^+^ CD25^+^ T cells were isolated from spleens with magnetic sorting and analysed by FCM (Fig. 7F/G). The CFSE fluorescence intensity was undetectable in the no treatment and 20 μg BN imDex treatment group. The percentage of divided CFSE-labelled Tregs was analysed. In the imDex/Tregs co-treatment group, the Treg proliferation rate was higher than in the Tregs treatment group (*p *= 0.0025, n = 3, Fig. 7G). These results suggested that imDex also amplifies Tregs *in vivo*.

## Discussion

Our results confirm that the combined use of proper doses of donor imDex and donor antigen-specific Tregs can induce rat liver allograft tolerance without the need for immunosuppressive agents. This active tolerance could be detected in cell mixing experiments *in vitro* (Fig. 5) and survival analysis/pathology analysis *in vivo* (Figs 3/4). Meanwhile, we found that exogenous Tregs were widely distributed in liver grafts, spleens, and mesenteric lymph nodes (Fig. 6) and that imDex could amplify Tregs. Recipient DCs were essential for this imDex function, and IL-2 was also helpful (Fig. 7). Fortunately, the expanded Tregs retained their regulatory ability and specificity, remaining tenable in the *in vivo* assay, which may explain the synergistic effect and the induction of tolerance by Tregs and imDex (Fig. 7).

ImDCs can inhibit immune rejection^20^,^21^, and exosomes have many advantages, including their stable nature and easy storage. We therefore added imDex to our treatment protocols to verify whether imDex can function similarly to imDCs in liver recipients. We found that the most effective imDex dosage (20 μg at one of three time points) prolonged the rat liver survival time, which is consistent with previous reports^11^. However, the optimal exosome dosage varies between studies and is not even described in some reports^6^,^22^; these differences may be due to the use of different animals, diverse models, various exosome sources or different dose gradient designs. In the *in vivo* assay, we verified that only donor-derived imDex (20 μg at one of three time points) prolonged recipient survival time, which is consistent with previous imDex studies^8^,^23^. However, this finding appears contradictory to those using DCs, as it was reported that infusion of either donor-20,24,25,26 or recipient-derived^27^,^28^ DCs with tolerogenic properties prolonged allograft survival time. Considering that there are at least two properties underlying the tolerogenic function of DCs, including “inherently tolerogenic properties” (clonal deletion, inhibition of T effector cells, and the expansion or induction of Tregs) and “negative cellular vaccines”^21^ (donor-derived tolerogenic DCs have donor antigen but do not induce rejection), we believe that donor-derived tolerogenic DCs with the “negative cellular vaccine” property may have some advantages and may work with relatively low cell numbers. Indeed, our results indicated that donor-derived imDex may also possess the “negative cellular vaccine” property. However, we did not compare imDex and mDex in our study, which may be a limitation.

After magnetic bead isolation and incubation with donor SDCs, the FOXP3^+^ rate slightly increased, consistent with previous studies^17^,^19^. However, the CD127^+^ rate decreased (Fig. 2A/B). While CD8a^+^ cytotoxic T cells play an important role in the cellular immune response to transplantation, we observed that SDC-expanded Tregs could inhibit these responses, including proliferation and the production of inflammatory cytokines, with donor specificity (Fig. 2C–E), indicating that SDC-expanded Tregs have the potential to inhibit the allograft rejection reaction. Although there are some controversies surrounding Treg specificity^29^,^30^,^31^, we found that donor-specific Tregs did prolong the liver allograft survival time in a donor-specific manner (Fig. 3C/Table 1). Therefore, we argue that donor specificity is important for Tregs in both the *in vitro* suppression response and the *in vivo* regulation of immune rejection for transplantation models.

Combined treatment with donor imDex and donor antigen-specific Tregs led to long-term survival (six of nine recipients in the co-treatment group survived over 100 d). However, in the untreated group, the MST was 10 d. In the imDex or Tregs treatment groups, the MST was 37 or 34 d, respectively (Fig. 3, Table 1). These results suggest that in the co-treatment group, acute rejection was delayed and relatively slight compared with the other groups and the findings of another study^32^. The pathological results of combined treatment also showed a relatively delayed and slight acute rejection (Fig. 4), and at 100 d after transplantation, we observed considerable regeneration. We then isolated total T cells from recipients and found that their proliferation and IFN-γ production were both reduced in a donor-specific manner. Together, these results demonstrate that allograft tolerance was achieved.

To explore exogenous Tregs distribution, we administered CFSE-labelled Tregs to recipients and found they were widely distributed in liver grafts, spleens and mesenteric lymph nodes. Interestingly, in the imDex/CFSE-labelled Tregs group, the number of exogenous Tregs was significantly higher than in the CFSE-labelled Tregs group (Fig. 6). We speculate that imDex amplified the exogenous Tregs, and this phenomenon may explain the synergistic effects in the co-treatment group. To verify this hypothesis, we performed *in vitro* Treg expansion assays (Fig. 7A/B). In these assays, imDex amplified Tregs only when SDCs were incubated in this system (Fig. 7A/B), confirming our and Morelli’s conjecture. Morelli reported^4^ that imDex works with DCs *in vivo* and speculated that this mechanism underlies the effects of imDex *in vivo*. These results also suggest that imDex expands Tregs in a similar manner to DCs, as donor DCs function in recipients with recipient DC assistance, and deletion of recipient DCs deters the therapeutic effect of donor DCs, as was reported by Wang. However, the mechanism of interaction between exosomes and assistant DCs has not been fully clarified. Genally speaking, there are two types of hypotheses: i) exosomes directly fuse with the membranes of assistant DCs or bind on the surface of DCs^33^,^34^,^35^, so that intact donor MHC molecules can present on recipient DCs and directly interact with effector cells by means of a “direct recognition pathway”^19^; ii) exosomes are endocytosed by recipient DCs, and donor MHC molecules and antigen peptides will be presented by assistant DCs^36^,^37^. In this way, exosomes only interact with effector cells by means of classic “indirect recognition pathway”. It remains unknown which pathway is dominant in donor imDex inducing recipient Tregs proliferative response.

Next, we found that IL-2 improved the ability of imDex to expand Tregs, which is similar to the DCs expanding assays reported by Yamazaki^16^ showing that IL-2 improved the ability of allogeneic DCs to amplify Tregs. Furthermore, we analysed the proliferation rate of CFSE-labelled Tregs *in vivo* and found that Treg proliferation increased with imDex treatment. This result confirms that imDex can expand Tregs both *in vitro* and *in vivo*. However, due to a lack of effective methods, we could not verify the mechanism of interaction between imDex and recipient DCs. We also did not explore whether imDex interacted with a wider range of cells.

In conclusion, imDex plays an immune regulatory role in rat liver transplantation. Our results identified the optimal dose for imDex administration and further verified that Treg antigen specificity is critical for immune regulation in allogeneic rat transplantation. Furthermore, the combined use of both imDex and Tregs induced liver allograft tolerance, and imDex amplified Tregs most likely through binding/fusing with recipient DCs and being presented by recipient DCs, which could be enhanced by IL-2. Synergistic effects between imDex and Tregs may explain why the co-treatment group survived longer than imDex or Tregs treatment groups. As liver allograft tolerance was induced and imDex possesses some advantages over imDCs, this study provides a new method for the regulation of transplantation immunity for clinical work.

## Methods

### Animals

Male BN RT1^n^, F344 RT1^lv^ and Lewis RT1^l^ rats (250–300 g) were purchased from Vital River, Inc. (Beijing, China). All rats were bred in a specific-pathogen–free animal facility. The research protocol was approved by the Animal Experiment Administration Committee of the Fourth Military Medical University and the research was carried out in accordance with the approved contents. Anaesthesia during liver transplantation and specimen procurement was maintained with ethyl ether, and all efforts were made to minimize suffering.

### Antibody

FITC-MHC Class I (OX18), FITC-MHC Class II (HIS19), PE-CD80 (3H5), PE-CD86 (24F), FITC-mouse IgG1 k isotype (P3.6.2.8.1), PE-mouse IgG1 k isotype (P3.6.2.8.1), PE-CTLA-4 (CD152, WKH203), FITC-ki67 (SolA15), FITC-ICOS (CD278, C398.4A), FITC-Rat IgG2a K Isotype (eBR2a) and FITC-Armenian hamster IgG isotype (eBio299Arm) antibodies were purchased from eBioscience (San Diego, CA, USA). A Rat Regulatory T Cell Multi-Color Flow Cytometry Kit (including FITC-CD4 (OX-38), PE-CD25 and APC-FOXP3 antibodies) was purchased from R&D (Minneapolis, MN, USA). Anti-rat CD127 (EPR2955(2)), Alexa Fluor 680-conjugated donkey anti-rabbit IgG and FITC-CD8 (MRC OX-8) antibodies were purchased from abcam (Cambridge, UK). Anti-rat-CD8a (G28) microbeads and anti-DC (OX62) microbeads were purchased from Miltenyi Biotech Inc. (Auburn, CA, USA).

### Isolation of CD4^+^ CD25^+^ T cells and CD8a^+^ T cells

CD4^+^ CD25^+^ cells were isolated from Lewis rat spleens with a MagCellect* Rat CD4^+^ CD25^+^ Regulatory T Cell Isolation Kit (R&D, Minneapolis, MN, USA, off the shelf now) according to instructions. Positive selection of CD8a^+^ cells from rat spleens was performed using anti-CD8a beads.

### Antigen-specific Tregs

Largely following Joffre’s description^38^, CD4^+^ CD25^+^ T cells (4 × 10^5^/well) from Lewis rats were co-cultured with 1 × 10^6^ SDCs (SDCs were enriched as described below) from BN/F344 rats in 24-well plates for 14 d. Cells were cultured in a final volume of 2 ml of T cell culture medium (RPMI 1640 supplemented with 10% FCS, 2 mM L-glutamine, penicillin, streptomycin, 10 mM HEPES, 50 μM 2-mercaptoethanol (2-ME), 1 mM nonessential amino acids, 1 mM sodium pyruvate, and 100 U/ml IL-2). On the 7^th^ day, 1 ml of fresh medium was added, and cells were cultured for another 7 d. After 14 d of co-culture, SDCs were depleted with anti-DC (ox62) beads, and 1−2 × 10^6^/well CD4^+^ CD25^+^ T cells were usually obtained.

### Bone marrow DCs and spleen DCs

DCs were generated from bone marrow (BM) cells as previously described^8^,^11^,^23^ with some modification. BM cells were cultured in complete medium (endotoxin-free, 10 ng/ml rat IL4 and 6 ng/ml murine GM-CSF were purchased from Peprotech (Rocky Hill, NJ, USA) for a total of 11 d. On the 6^th^ day, the medium was refreshed while the original medium supernatant was harvested. From then on, some of the cells were cultured in medium containing LPS (100 ng/ml, Sigma, St. Louis, USA) for 1 d and the medium was then refreshed. At the end of 11 d culture, immature DCs, mature DCs and the corresponding supernatants were harvested. Spleen DCs were isolated from donors or recipients with anti-DC (ox62) beads as per the instructions.

### Exosome preparation

Exosomes were isolated from mDC and imDC supernatants (imDC supernatants at 6 and 11 d were pooled to obtain greater output) using an exosome isolation kit (Life Technologies, Carlsbad, California, USA) according to the manufacturer’s instructions. The pellet was then re-suspended with saline. Approximately 15 μg of exosomes were harvested from 1 × 10^7^ mDCs or imDCs. The exosome amount was evaluated based on the amount of protein using the Bradford assay. Then, exosomes were stored at −80 °C for future use.

### Flow cytometry

As previously described^8^, except that aldehyde/sulfate latex beads were purchased from Thermo Fisher Scientific (Waltham, MA, USA). The beads attached to the two types of exosomes were stained with FITC-MHC class I, FITC-MHC class II, PE-CD80 or PE-CD86 antibodies. Beads stained with FITC and PE isotype antibodies formed the control groups. CD4^+^ CD25^+^ T cells and CD8a^+^ T cells were stained with FITC-CD4, PE-CD25, APC-FOXP3, unconjugated CD127, Alexa Fluor 680-conjugated donkey anti-rabbit IgG and FITC-CD8 antibodies as described in the product specifications. Then, the beads or cells were assessed by FCM using a FACSCalibur (BD Immunocytometry Systems, Franklin Lakes, NJ, USA), and data were analysed with FlowJo 7.6.

### Electron microscopy

As described by Meckes^39^, the sample was diluted ten times, loaded onto a copper grid at room temperature, and examined with a CM120 Philips Biotwin electron microscope (Phillips Electronic Instruments).

### Orthotopic liver transplantation, infusion therapy and survival time recording

Surgical procedures were performed as Kamada^40^ described. In this procedure, we used BN rats as liver donors, F344 rats as other allogenic donors and Lewis rats as allograft recipients. Cuffs were used. No immunosuppression was given to recipient rats in this study. Different doses of imDex and/or exogenous Tregs (2 × 10^6^ per time-point), either unlabelled or labelled with 10/20 μM CFSE (eBioscience, San Diego, CA, USA), were transferred via caudal injection 7 d before, the day of, and 7 d after transplantation. Recipients that died within 3 d were regarded as technical failures and excluded from further analysis. On day 0, 10, 35 and 100 after transplantation, three recipients were sacrificed for HE analysis and/or anti-donor cellular response assays. These animals were excluded from the survival analysis. If needed, extra transplantation was carried out to ensure 9 recipients in each group. Recipient survival times were recorded and analysed, and the experiment ended after 100 d or 60 d.

### Mixed leukocyte reactions

In the Treg suppression assay, Fresh Tregs, Lewis-derived BN/F344 antigen-specific Tregs and CFSE (5 μM)-labelled CD8a^+^ T cells were obtained and co-cultured at various Treg/CD8a^+^ T cells ratios (BN SDCs as stimulator cells, irradiated by 20 Gy/2,000 rad γ-ray) in 200 μl of T cell medium in a 96-well round-bottom plate at 37 °C. Starting cell numbers were 5 × 10^4^ Tregs, 5 × 10^4^ CFSE-labelled CD8a^+^ T cells, and 5 × 10^4^ stimulator cells. Different numbers of Tregs were added later. Five days later, CFSE intensity was measured by FCM, and the rate of divided CFSE-labelled cells was analysed with FlowJo 7.6 and recorded as proliferation rate. The expanded Treg suppression assays were carried out as mentioned above, except that the expanded Tregs were used as suppressor and BN/F344 SDCs were used as stimulator. In the recipient leukocyte proliferation assay, as described by Peche^11^, 5 × 10^4^ Lewis total T cells, purified from recipients’ spleencytes by Rat T Cell Enrichment Column kit (R & D, Minneapolis, MN, USA), were labelled with 5 μM CFSE and were added to 96-well round-bottom plates with equal numbers of irradiated SDCs derived from BN/F344. Five days later, CFSE intensity was measured and analysed as mentioned above. The IFN-γ levels in this culture supernatant were determined using the Rat IFN-γ ELISA kit purchased from NeoBioscience (Beijing, China).

### Histological examinations

Liver grafts harvested on day 0, 10, 35 and 100 after transplantation were fixed for 72 h, embedded in paraffin, sectioned at 6 μm and stained with HE for histological examination. The RAI according to the Banff schema^32^,^41^ was used to evaluate allograft rejection. An independent test was designed with CFSE-labelled Tregs (20 μM CFSE, 10 min at room temperature) in which 12 extra recipients received liver transplantation and four different treatments (no treatment, 20 μg of imDex, CFSE-labelled BN-specific Tregs or imDex/CFSE-labelled Tregs co-treatment. Each group contained 3 recipients.). On the 10^th^ day after transplantation, sample fragments were snap-frozen in OCT (Tissue-Tek, Elkhardt, IN, USA), cut into 8-μm sections, and stained with DAPI.

### Treg proliferation assays *in vitro* and *in vivo*

We cultured 1 × 10^4^ Lewis-derived BN-specific Tregs (5 μM CFSE labelled) in 96-well plates and incubated them with 0.5 μg of BN imDex, 2 × 10^4^ Lewis SDCs and/or IL-2 (200 units/ml, recombinant rat IL-2 purchased from Peprotech, Rocky Hill, NJ, USA). Seven days later, proliferation was analysed. In the *in vivo* assays, Tregs were labelled with 10 μM CFSE. After 10 min at room temperature, the reaction was stopped by the addition of an equal volume of FCS, followed by washing in PBS. A total of 2 × 10^6^ Tregs were injected as described above. Ten days after transplantation, CD4^+^ CD25^+^ T cells were isolated from recipient splenocytes by magnetic sorting. Gated on the CD4^+^ CD25^+^ CD127^−^ CFSE^+^ cell subset, the percentage of divided CFSE-labelled Tregs was analysed.

### Statistics

The data points in the line graphs and bar graphs represent the mean ± SEM or mean + SEM, respectively. Data were expressed as the means ± standard error of means (SEM) and analysed using Student’s two-tailed t test for comparison between two groups, One Way ANOVA for comparison among more than two groups or the log-rank test for survival analysis. Values were analysed using GraphPad Prism 5.0 (GraphPad, San Diego, CA, USA). The results were deemed statistically significant if the *p*-value was <0.05. Statistical significance is indicated in each figure where applicable.

## Additional Information

**How to cite this article**: Ma, B. *et al.* Combining Exosomes Derived from Immature DCs with Donor Antigen-Specific Treg Cells Induces Tolerance in a Rat Liver Allograft Model. *Sci. Rep.* **6**, 32971; doi: 10.1038/srep32971 (2016).

## Supplementary Material

Supplementary Information

## Figures and Tables

**Table 1:** Recipient survival after treatment with imDex and/or Tregs (log-rank test).

Treatment	Median survival (days)	Individual Survival (days)	*p* value
Untreated△	10	6 × 2, 9 × 2, 10, 11, 13, 16 × 2	
1 μg imDex BN	10	7, 8, 9 × 2, 10, 12 × 2, 13, 14	=0.5419^a^
10 μg imDex BN	17	9 × 3, 12, 17 × 2, 19, 21 × 2	=0.0241^a^, =0.0087^b^
20 μg imDex BN	37	14, 17 × 2, 21, 37, 47, 49, 52, 53	<0.0001^a^, =0.007^d^
40 μg imDex BN	25	12, 15, 16, 19, 25, 29, 31 × 2, 33	= 0.0008^a^, =0.0498^b^
80 μg imDex BN	26	12, 14 × 2, 21, 26, 27 × 2, 29, 31	=0.0011^a^, =0.0441^b^
20 μg imDex Lewis	14	7 × 2, 8, 10, 14 × 2, 17 × 2, 19	=0.1754^a^, =0.0011^b^
20 μg imDex F344	15	7, 9, 10, 14, 15, 16 × 2, 24, 25	=0.1159^a^, =0.0041^b^
Tregs fresh	18	6, 8, 14, 18 × 2, 24 × 2, 31, 32	=0.0081^a^, =0.01^c^
Tregs BN-specific	34	17, 18, 27, 29, 34 × 2, 42, 45, 47	<0.0001^a^, =0.0083^d^
Tregs F344-specific	19	11 × 2, 13, 15, 19 × 2, 26, 27 × 2	=0.006^a^, =0.00321^c^
20 μg imDex BN/Tregs fresh	47	14 × 2, 16, 36, 47, 49, 56, 58, 60#	=0.0005^a^, =0.283^b^, =0.0386^d^
20 μg imDex BN/Tregs BN-specific	>100 undifined	28, 31, 33, 100# × 6	<0.0001^a^

Lewis recipients were transplanted with BN livers. Recipients were treated with imDex and/or Tregs.

^Δ^Treated with physiological saline.

^a^Compared with untreated rats.

^b^Compared with 20 μg imDex BN.

^c^Compared with Lewis Tregs BN-specific.

^d^Compared with 20 μg imDex BN/Lewis Tregs (BN-specific).

^#^Not dead on the day we analyzed data.
