# Supplementary material for: Combining Exosomes Derived from Immature DCs with Donor Antigen-Specific Treg Cells Induces Tolerance in a Rat Liver Allograft Model
Source: Sci Rep. 2016 Sep 19;6:32971. doi: 10.1038/srep32971 (PMC5027549; doi:10.1038/srep32971)
Supplement: Supplementary Information [file srep32971-s1.pdf]

Supplementary Information

Combining Exosomes Derived from Immature DCs with Donor Antigen-Specific Treg Cells Induces Tolerance in a Rat Liver Allograft Model

Ben Ma[1△], Jing-Yue Yang[2△], Wen-jie Song[1△], Rui Ding[1△], Zhuo-chao Zhang[1], Hong-chen Ji[1], Xuan Zhang[1], Jian-lin Wang[1], Xi-sheng Yang[1], Kai-shan Tao[1*], Ke-feng Dou[1*], Xiao Li[1*]

1 Department of Hepatobiliary Surgery, Xijing Hospital, Fourth Military Medical University, Xi'an, Shaanxi Province, China.

2 Department of Clinical Oncology, Xijing Hospital, Fourth Military Medical University, Xi'an, Shaanxi Province, China

△ These authors contributed equally to this work.

Correspondence should be addressed to XL (lixiao0076@163.com), K-F D (gdwkgwx@fmmu.edu.cn) or K-S T (taokaishan0686@163.com).

The authors declare no competing financial interest.

Supplementary Table. S1. Phenotypic features of different fractions of isolated T cells

**Sample**	**% $CD4^+CD25^+$**	**% $CD4^+CD25^+FOXP3^+$**	**% CD4/CD25/CD127**
A1	91	69.4	14.9
A2	83.5	63.1	10.1
A3	92.9	73.1	13.7
MST/SEM	89.1±2.9	68.5±2.9	12.9±1.4
B1	92.2	88.2	7.1
B2	87.8	81.3	5.5
B3	93.1	91.3	9.1
MST/SEM	91.0±1.6	86.9±3.0	7.2±1.0
**Sample**	**% $CD8^+$**		
C1	88.7		
C2	84.9		
C3	93.8		
MST/SEM	89.1±2.6		

Samples A: fresh isolated $CD4^+CD25^+$ T cells

Samples B: donor SDCs co-cultured $CD4^+CD25^+$ T cells

Samples C: isolated $CD8a^+$ T cells

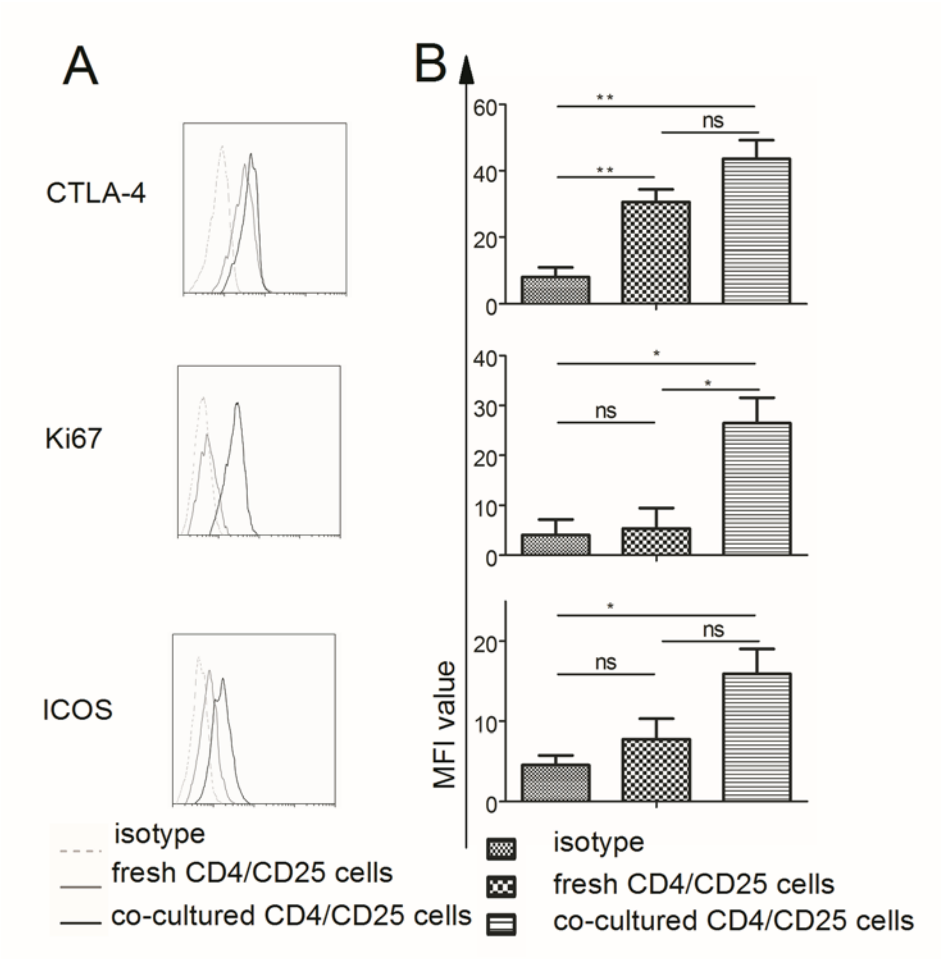

Supplementary Fig. S1. Additional phenotype identification of Treg. (A) To further identify the statue of two fraction of Treg (fresh isolated Tregs and Tregs co-cultured with donor SDCs), we detected the expression of CTLA-4, Ki67 and ICOS with FCM. One representative result of three is shown. (B) Mean fluorescence intensity (MFI) was analysed. Ki67 expression was higher in co-cultured Tregs than fresh isolated Tregs, $p < 0.05$, n = 3. * indicates $p < 0.05$; ** indicates $p < 0.01$; ns indicates no significant difference.

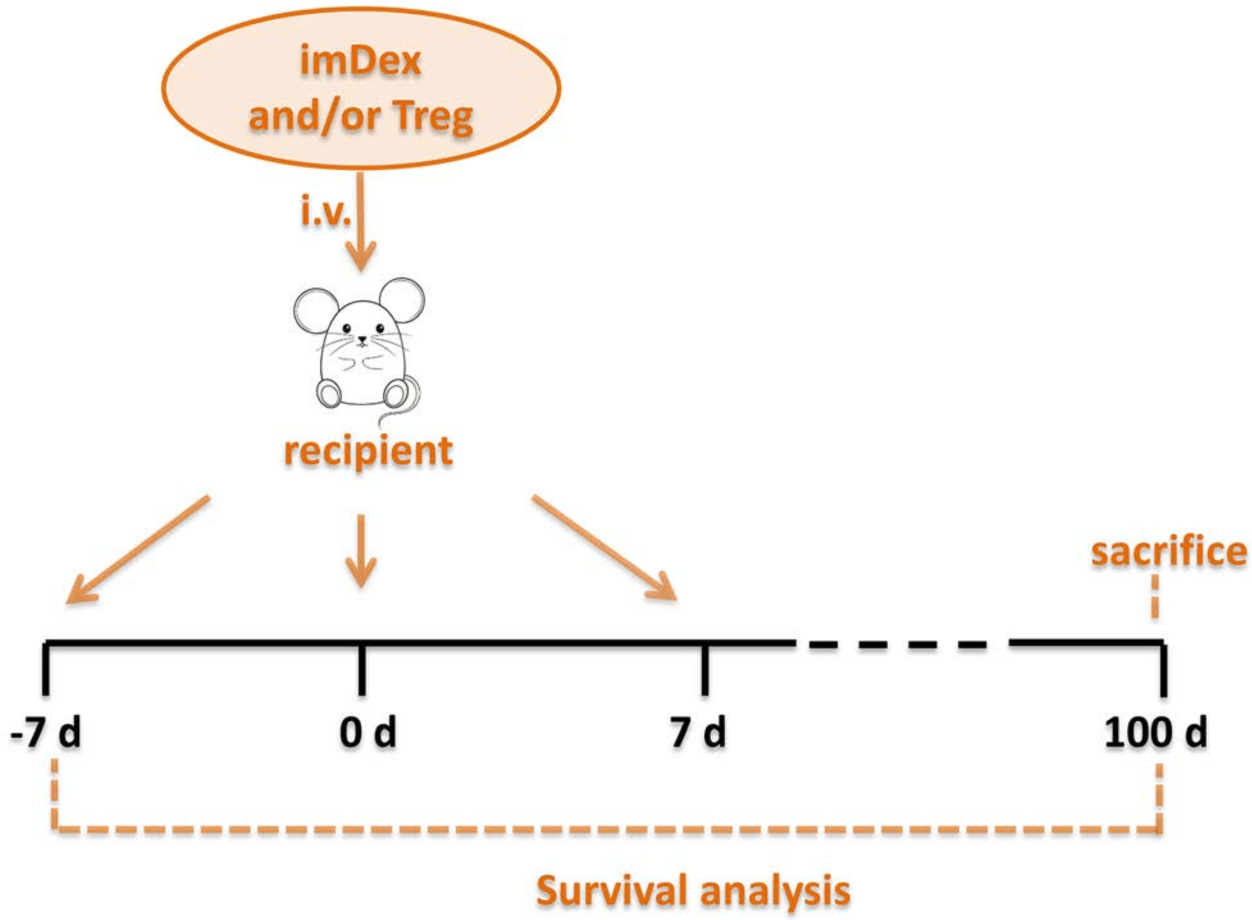

Supplementary Fig. S2. Process diagram of survival analysis in fig. 3.

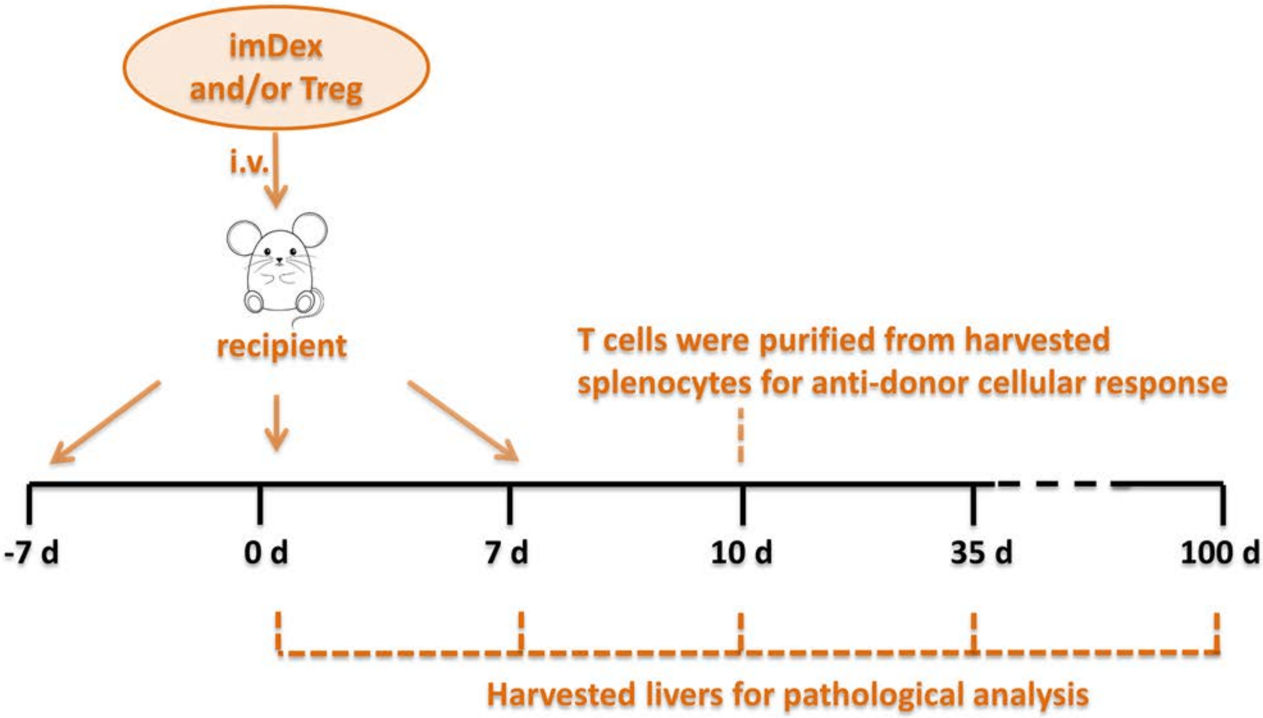

Supplementary Fig. S3. Process diagram of anti-donor cell response analysis and pathological analysis in fig. 4/5.

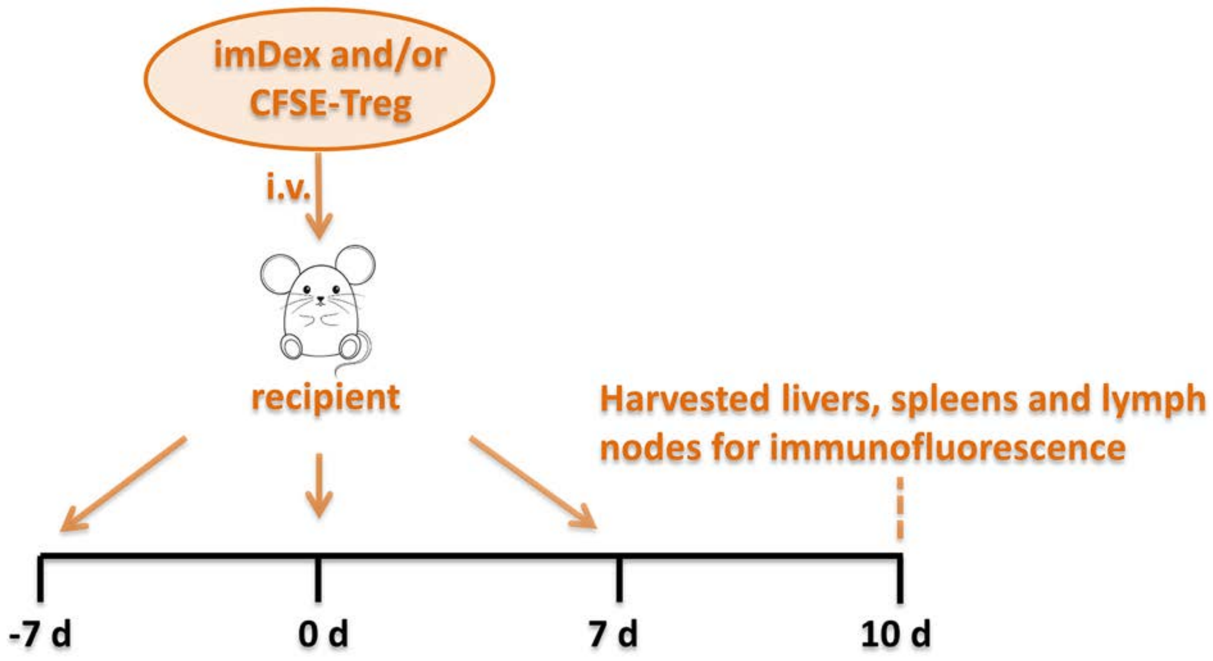

Supplementary Fig. S4. Process diagram of immunofluorescence analysis in fig. 6.

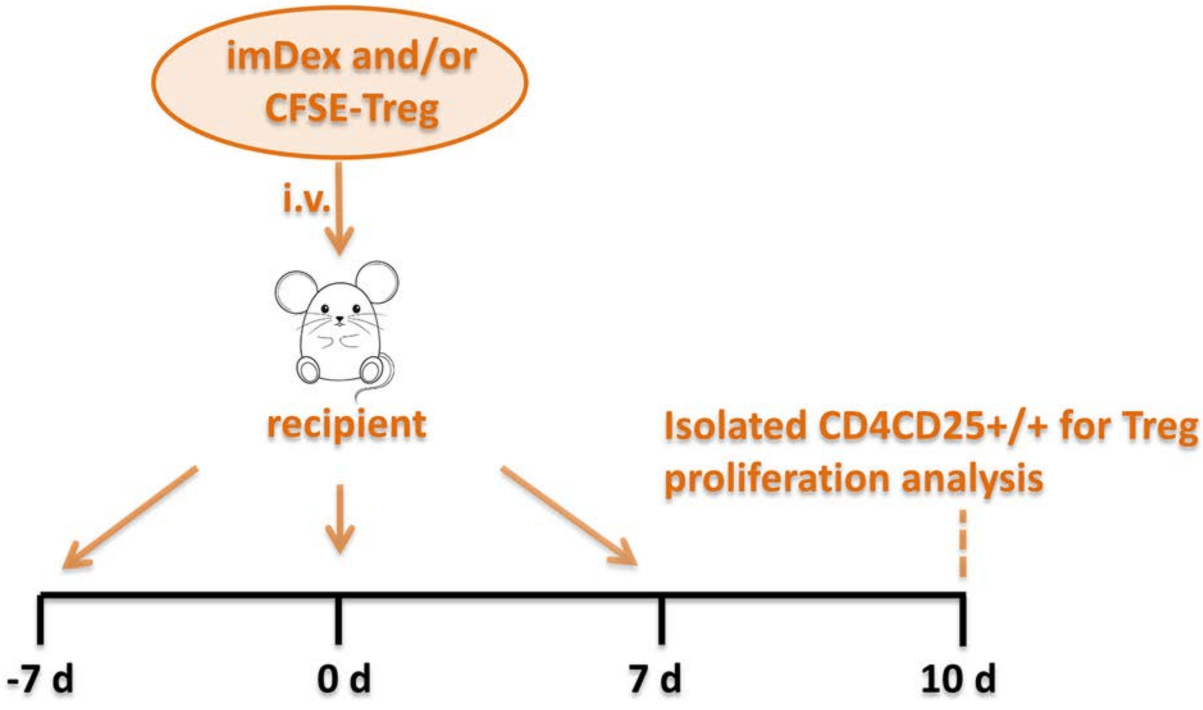

Supplementary Fig. S5. Process diagram of donor-specific Treg proliferation assays in vivo in fig. 7.
